# Endotoxemia‐Induced Inflammation in the Absence of Obesity Is Associated With Decreased Anxiety‐Like and Impulsive Behavior With no Effect on Learning and Memory

**DOI:** 10.1002/cph4.70044

**Published:** 2025-08-29

**Authors:** Molly E. Klug, Eden Crain, Anna M. R. Hayes, Mugil V. Shanmugam, Claire B. de La Serre, Scott E. Kanoski

**Affiliations:** ^1^ Human and Evolutionary Biology Section, Department of Biological Sciences, Dornsife College of Letters, Arts and Sciences University of Southern California Los Angeles California USA; ^2^ Department of Nutritional Sciences University of Georgia Athens Georgia USA; ^3^ Department of Food Science and Technology, College of Agricultural Sciences Oregon State University Corvallis Oregon USA; ^4^ Department of Biomedical Sciences Colorado State University Fort Collins Colorado USA

## Abstract

Obesity is associated with increased gut permeability, which contributes to a state of chronic low‐grade inflammation. Obesity is also linked with altered neurocognitive functions, including impaired learning and memory. Whether these changes are secondary to neuroinflammation versus other comorbidities associated with obesity is unknown. Here, we modeled the chronic low‐grade inflammation that accompanies diet‐induced obesity, but in the absence of obesity or consumption of an obesogenic diet. Male rats were implanted with intraperitoneal osmotic minipumps, continuously dispensing either saline (control) or lipopolysaccharide (LPS), an endotoxin produced in the gut that triggers inflammation when in circulation. Immunohistochemistry results revealed that LPS exposure led to neuroinflammation, with an increased number of Ionized Calcium‐Binding Molecule 1 (Iba1^+^) cells in the amygdala and hippocampus in LPS rats. Given that these brain regions are associated with impulse control, anxiety‐like behavior, and learning and memory, we tested whether chronic LPS treatment impacted these behaviors. Interestingly, LPS exposure did not affect hippocampal‐dependent memory in the Morris water maze, novel location recognition, or novel object in context memory tests, suggesting that neuroinflammation in the absence of obesity does not induce memory impairments. Further, chronic LPS significantly decreased anxiety‐like behavior in the open field test and food impulsivity in an operant‐based procedure. LPS animals also had significantly lower corticosterone and melatonin levels compared to controls, which may contribute to these behavioral outcomes. These results suggest that the low‐grade inflammation associated with obesity is not sufficient alone to drive obesity‐associated memory impairments but does reduce anxiety and food‐motivated impulsive responses.

## Graphical abstract

Obesity is associated with chronic inflammation and altered neurocognitive functions, but whether these outcomes are functionally linked is unknown. To model the inflammation accompanying obesity, minipumps dispensing the endotoxin lipopolysaccharide (LPS) led to neuroinflammation, decreases in anxiety‐like and impulsive behaviors, yet had no effect on hippocampal‐dependent memory relative to controls.

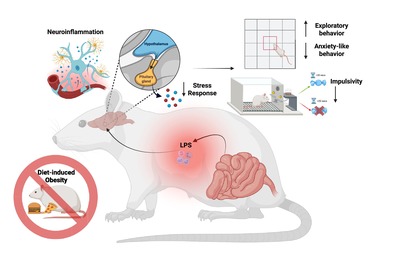

## 1 Introduction

Obesity is a growing health concern, with approximately 40% of US adults now classified as obese (CDC 2024). Consumption of a high‐fat diet and obesity are each associated with a host of negative health outcomes, including increased gut permeability (i.e., a “leaky gut”) (Moreira et al. 2012; Mishra et al. 2023; Rainone et al. 2016; Nagpal et al. 2018). One consequence of diet‐ and/or obesity‐associated leaky gut is metabolic endotoxemia, in which higher levels of bacterial‐derived lipopolysaccharide (LPS) in circulation contribute to a state of chronic low‐grade inflammation (Cani et al. 2007; Fuke et al. 2019; Pendyala et al. 2012). LPS is a component of the outer membrane of Gram‐negative bacteria, which are produced abundantly in the gut and initiate inflammatory responses via Toll‐like receptor 4 activation (Cani et al. 2007; Fuke et al. 2019). With obesity‐associated increases in intestinal permeability (Cani et al. 2008), LPS leaks into circulation and promotes peripheral inflammation (Cani et al. 2007; Cani et al. 2008). This endotoxemia‐induced peripheral inflammation can lead to blood–brain barrier damage and neuroinflammation (Brown 2019; Peng et al. 2021; Guillemot‐Legris and Muccioli 2017), thus representing a putative mechanism through which unhealthy diets and obesity contribute to a range of neurocognitive deficits (Anand et al. 2022; Smith et al. 2011).

Learning and memory processes that require the hippocampus (HPC) are particularly vulnerable to disruption by obesity and by Western diet (high in fat, sugar, and processed foods) consumption in the absence of obesity (Cordner and Tamashiro 2015; Kanoski and Davidson 2011; Noble et al. 2017; Noble et al. 2021; Hayes, Lauer, et al. 2024; Francis and Stevenson 2013). Both obesity and Western diet consumption are also associated with altered anxiety‐like behavior (Fulton et al. 2022), reward‐motivated behaviors (Volkow et al. 2011; Wallace and Fordahl 2022), and impulsivity (Shi et al. 2022; Lavagnino et al. 2016; Giel et al. 2017), yet these relationships are complex, potentially bidirectional, and poorly understood. Additionally, the neuroinflammation associated with obesity and Western diet consumption can also impact the above cognitive domains (de Paula et al. 2021; Zheng et al. 2021), further complicating understanding of causal relationships linking obesity with cognitive outcomes.

In addition to neuroinflammation, obesity is linked with various other physiological changes that may impact cognitive function. Disentangling the myriads of possible contributing factors requires isolating them and evaluating cognitive outcomes in the absence of obesity or consumption of a Western diet. Here, we model the LPS endotoxemia‐induced chronic systemic inflammation associated with increased gut permeability in otherwise lean rats maintained on a healthy diet. Further, this model induces neuroinflammation in brain regions linked with cognitive processes impacted by obesity, thus offering a platform to evaluate the causal role of endotoxemia‐induced neuroinflammation in the absence of obesity on neurocognitive outcomes.

## 2 Materials and Methods

### 2.1 Animals and Housing

Male Wistar rats (~200 g) were obtained from Envigo (Indianapolis, IN, USA) and single‐housed in a temperature‐controlled 12‐h light/dark cycle facility. Animals were provided ad libitum access to standard rodent chow (Laboratory Rodent Diet 5001, LabDiets, St. Louis, MO, USA) and water. Body weight and food intake were measured at least three times per week. All procedures were approved by the Institutional Animal Care and Use Committees of the University of Georgia and the University of Southern California.

### 2.2 Surgery

Multiple cohorts were used for this study. For cohort 1, surgeries took place after several days to habituate to the facilities. For cohorts 2 and 3, surgery took place following behavioral training in either the differential reinforcement of low rates of responding (DRL) or progressive ratio (PR) operant tasks. During surgery, animals were implanted intraperitoneally with mini osmotic pumps (#2006, Alzet, Cupertino, CA, USA) containing either sterile saline (0.9% NaCl) or an LPS solution providing 12.5 μg/kg/h (LPS isolated from *Escherichia coli* O26: B6, #L3755, Sigma Aldrich) to be delivered for 6 weeks. The LPS dose was based on previous studies (Cani et al. 2007; de La Serre et al. 2015). To prepare for surgery, rats were shaved on the lower left quadrant of the abdomen, and the surgical site was sterilized with betadine. Surgery was performed under isoflurane inhalation anesthesia at a dose of 2%–3%. Animals were injected with carprofen or ketoprofen (subcutaneous, 5 mg/kg for both) preoperatively for analgesia. A 2 cm incision was made, and pumps were implanted intraperitoneally, after which the skin and muscle layers were sutured together and closed with sutures or wound clips. Animals were given topical antibiotic postoperatively. Wound clips or stitches were removed 7–10 days post‐surgery.

### 2.3 Behavior Testing

**FIGURE 1:**
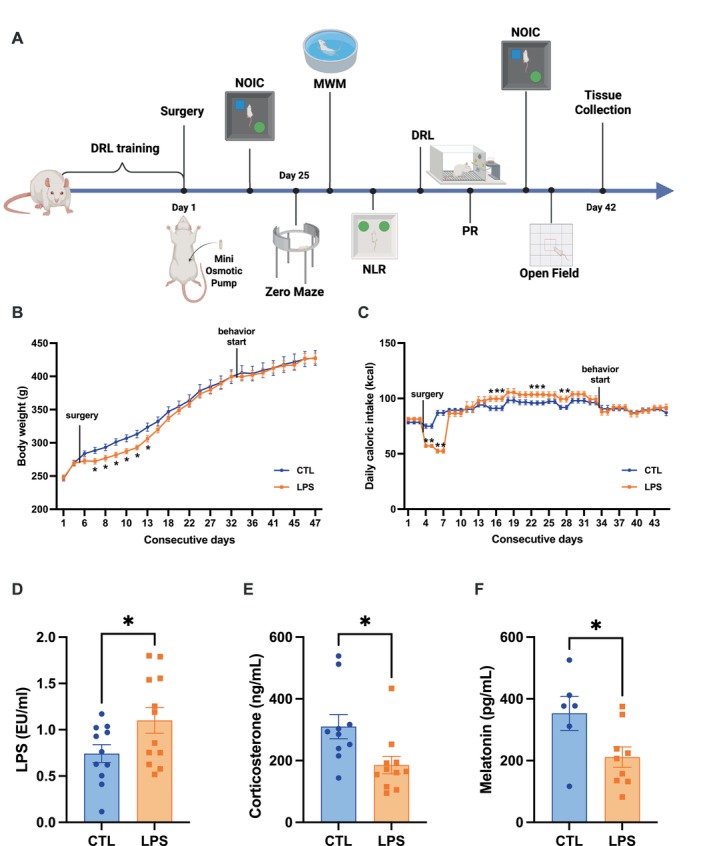
Chronic low‐dose LPS administration increases food intake and serum LPS levels, without affecting long‐term body weight. (A) Experimental timeline (not drawn to scale). Chronic LPS administration causes initial, post‐surgical weight loss but does not affect long term body weight (B). Similarly, chronic LPS administration causes an initial decrease in food intake, followed by increases in food intake before intake ultimately stabilizes (C). Chronic LPS administration increases serum LPS (D), and decreases serum corticosterone (CORT) (E) and melatonin (F) when compared to controls. (LPS *n* = 10–12, CTL *n* = 10–11 for BW, FI, Serum LPS and CORT; LPS = 9, CTL = 6 for melatonin; all between‐subjects design; data are means ± SEM; *p < 0.05, **p < 0.01 ***p < 0.001).

Behavior testing started 4 weeks after surgery as previous research has shown that metabolic endotoxemia is fully developed after 4 weeks (Cani et al. 2007). Animals received at least 1 day of rest between different behavioral tests, and behavioral testing was completed by 6 weeks post‐surgery. Hippocampal‐dependent spatial learning and memory was tested using the Morris water maze (MWM) (28 days post‐surgery (PS)), novel location recognition (NLR) (30–34 days PS), and novel object in context (NOIC) tests (21–23, 37–39 days PS). One hippocampal‐dependent memory test (NOIC) was tested at two different timepoints to evaluate whether differential results would be obtained based on the time since minipump implantation. Impulsive responding, food reward effort‐based responding, and anxiety‐like and exploratory behavior were assessed using the differential reinforcement of low rates of responding (DRL) task (impulsivity) (35 days PS), the progressive ratio (PR) reinforcement schedule operant task (food reward motivation) (36 days PS), the Zero Maze (ZM) task (25 days PS) and the open field test (OFT) (anxiety‐like and exploratory behaviors) (36 days PS). A general timeline of experimental procedures is shown in Figure 1A. Behavioral tests were conducted in 3 separate cohorts: Cohort 1 was tested in MWM, NLR, and OFT. Cohort 2 was tested in NOIC, DRL, and ZM. Cohort 3 was tested in PR.

#### 2.3.1 Morris Water Maze (MWM)

The MWM task, a test of hippocampal‐dependent spatial reference memory (D'Hooge and Deyn 2001), was conducted over 4 days of training with a final memory probe test on Day 5. Training and testing were done in a blue pool (diameter 170 cm) filled with room‐temperature water. A resting platform (diameter 10 cm) to escape swimming was placed in the center of one quadrant, and large, high‐contrast visual cues were placed on the walls surrounding the pool. The platform and cues remained in the same positions for the duration of the experiment. On each training day, animals were placed in the pool and allowed to swim for 60 s or until the platform was found. If they had not found the platform in 60 s, they were guided to the platform and allowed to stay for 15 s. Each rat received five trials per training day with approximately 15‐min intervals between trials. After day one, the water was clouded with non‐toxic paint, and the platform was submerged 1 cm below the surface of the water. Rats were started in the same quadrant on days one and two, after which the start quadrant was varied by day. On the test day, the platform was removed, and animals were started in the same quadrant as training days one and two. Rats were allowed to swim for 60 s before being removed from the pool. The test was recorded with a video camera and analyzed using ANY‐maze software to determine swimming patterns and locations (Stoelting Co., Wood Dale, IL).

#### 2.3.2 Novel Location Recognition (NLR)

The NLR test was conducted using a 50 × 50 × 50cm opaque plexiglass arena with patterned markings on the walls to allow the rats to recognize their location. The test was conducted in dim lighting, and light covering was approximately equal between the center and corners. Rats were habituated to the box for 10 min a day for four consecutive days prior to the test day. On test day, two identical objects were placed in parallel corners of the box and animals were allowed to explore for 5 min. After a 5‐min interval, animals were returned to the box for 3 min with one of the objects moved to a new location. The object moved was counterbalanced between animals and experimental groups. Boxes and objects were cleaned between animals and trials with 10% ethanol. The test was recorded with a video camera and scored manually by an experimenter blinded to the treatments. For scoring, exploratory behavior was recorded for each object and was defined as follows: nose within 2 cm of the object, oriented towards the object or touching the object; not pointing nose into the air, grooming, or sitting on object (unless nose oriented towards object). Exploration was recorded for the initial 5‐min trial to assess baseline exploratory behavior and was recorded for the subsequent 3‐min trial for each object. Objects were defined as “Novel Location Object” and “Familiar Location Object” and “Total Exploration Time” for the test phase was calculated as the sum of the time spent exploring both the Novel and Familiar Location objects. The ‘Discrimination Index’ was calculated as follows: (time exploring Novel Location Object/Total Exploration Time) × 100%. The test is based on the rodent preference for novelty and assumes that, if the rodents accurately remember the location of the objects, they will favor the novel location object for exploration over the familiar location object. The use of location and contextual cues ensures that this test requires the HPC (Broadbent et al. 2004; Clark et al. 2005; Kendig et al. 2019).

#### 2.3.3 Novel Object in Context (NOIC)

NOIC was conducted to assess HPC‐dependent contextual episodic memory (Balderas et al. 2008; Martínez et al. 2014). The 5‐day NOIC procedure was adapted from previous research (Davis et al. 2020; Noble et al. 2021; Hayes, Lauer, et al. 2024; Suarez et al. 2018; Tsan, Chometton, et al. 2022; Hayes et al. 2022). Each day consisted of one 5‐min session per animal, with cleaning the apparatus and objects with 10% ethanol between each animal. Days 1 and 2 were habituation to the contexts—rats were placed in Context 1, a semitransparent box (41.9 cm L × 41.9 cm W × 38.1 cm H) with yellow stripes, or Context 2, a black opaque box (38.1 cm L × 63.5 cm W × 35.6 cm H). Each context was presented in a distinct room, both with similar dim ambient lighting yet with distinct extra‐box contextual features. Rats were exposed to one context per day in a counterbalanced order per treatment group for the two habituation days.

Following these two habituation days, on the next day each animal was placed in Context 1 containing a single copy of Object A and Object B situated on diagonal equidistant markings with sufficient space for the rat to circle the objects (NOIC Day 1). Objects were an assortment of hard plastic containers, tin canisters (with covers), and the Original Magic 8‐Ball (objects were distinct from what animals were exposed to in NLR). The sides where the objects were situated were counterbalanced per rat by treatment group. On the following day (NOIC Day 2), rats were placed in Context 2 with duplicate copies of Object A. The test day (NOIC Day 3) took place 1 day after NOIC Day 2, during which rats were again placed in Context 2, but with single copies of Object A and Object B; Object B was not a novel object per se, but its placement in Context 2 was novel to animals. Rats were consistently placed with their head facing away from both objects when placed into the training and test contexts. On NOIC Days 1 and 3, object exploration, defined as the rat sniffing or touching the object with the nose or forepaws, was quantified by hand‐scoring of videos by an experimenter blinded to the animal group assignments. The discrimination index for Object B was calculated for NOIC Days 1 and 3 as follows: time spent exploring Object B (the “novel object in context” in Context 2)/[time spent exploring Object A + time spent exploring Object B]. Data were then expressed as a percent shift from baseline as: [Day 3 discrimination index—Day 1 discrimination index] × 100. Rats with intact HPC function will preferentially explore the “novel object in context” on NOIC Day 3, while HPC impairment will impede such preferential exploration (Balderas et al. 2008; Martínez et al. 2014).

#### 2.3.4 Differential Reinforcement of Low Rates of Responding Task (DRL)

As previously described (Noble et al. 2019), non‐food restricted rats were trained in the early nocturnal phase on an operant lever‐pressing task to obtain a single, high‐fat high‐sugar 45 mg pellet (HFHS; F05989, Bio‐serv; 35% kcal fat and sucrose‐enriched) for each press. Training and test sessions were conducted in operant conditioning boxes (Med Associates Inc., St. Albans, VT). After progressively increased delay periods (0‐, 5‐, 10‐s delays, 5 days of training for each) over 4 weeks of training, rats were given a “DRL‐20” schedule for 10 days in which they must wait 20 s between lever presses. A lever press prior to the conclusion of the 20 s interval resets the clock, is not reinforced, and is considered an impulsive response. Training took place prior to the implantation of the mini osmotic pump to ensure that learning of the DRL task was unaffected by experimental treatment. After surgery, rats were trained 2×/week, separated by at least 1 day, at the DRL20 schedule. A single test day took place at the DRL20 schedule 5 weeks after the pump implantation.

#### 2.3.5 Progressive Ratio Task (PR)

Rats were trained to lever press for 45 mg pellet (HFHS; F05989, Bio‐serv; 35% kcal fat and sucrose‐enriched) in operant conditioning boxes over the course of 6 days, with a 1 h session each day. The first 2 days consisted of fixed ratio 1 with autoshaping, wherein animals would receive 1 pellet for each correct lever press (FR1), and a pellet would dispense automatically every 10 min. The next 4 days consisted of FR1 without autoshaping (2 days) and FR3 (2 days), wherein animals would receive 1 pellet for every 3 presses on the active lever. Training took place prior to the implantation of the mini osmotic pump to ensure that learning of the task was unaffected by experimental treatment. Post surgery, animals were periodically trained at a FR3 reinforcement schedule. Testing took place roughly 5 weeks after pump implantation. On the test day, rats were placed back in the operant chambers to lever press for HFHS pellets under a progressive ratio reinforcement schedule. The response requirement increased progressively using the following formula: F(i) = 5eˆ0.2i‐5, where F(i) is the number of lever presses required for the next pellet at i = pellet number, and the breakpoint was defined as the final completed lever press requirement that preceded a 20‐min period without earning a reinforcer, as described previously (Décarie‐Spain et al. 2022; Hsu et al. 2015).

#### 2.3.6 Zero Maze (ZM)

Following established procedures (Hayes, Lauer, et al. 2024), the zero maze apparatus was used to examine exploratory and anxiety‐like behavior. The apparatus consisted of an elevated circular track (11.4 cm wide track, 73.7 cm height from track to ground, 92.7 cm exterior diameter) that is divided into four equal‐length segments: two sections with 3‐cm high curbs (open) and two sections with 17.5‐cm height walls (closed). Ambient lighting was used during testing. Rats were placed in the maze on an open section of the track and allowed to roam freely for 5 min. The apparatus was cleaned with 10% ethanol between rats. The total distance traveled and time spent in the open segments of the apparatus were measured via video recording using ANY‐maze activity tracking software.

#### 2.3.7 Open Field Test (OFT)

The OFT to assess exploratory and anxiety‐like behavior was conducted using a 60 × 60 × 40cm clear plexiglass box in brightly lit (~200 lux) conditions. The arena was divided into nine 20 × 20cm sections. Animals were placed in the box for 10 min, and the box was cleaned between animals with 10% ethanol. The test was recorded with a video camera, and video footage was analyzed for the first 2 min using ANY‐maze software. For analysis, the center zone was defined as the 20 cm square in the middle of the arena, and corner zones were defined as each of the four corners. Behavior was analyzed for total distance, time in the center zone, and corner zones, and number of center zone entries.

### 2.4 Serum Corticosterone, LPS, and Melatonin

Serum LPS and corticosterone levels were measured in Cohort 1, and serum melatonin and cytokines were measured in Cohort 2. For serum corticosterone (CORT) and LPS measurements, animals were anesthetized at 6 weeks post‐surgery via CO_2_ inhalation. Blood was collected via cardiac puncture, allowed to clot on ice for 30 min, and centrifuged for 10 min at 4°C. Timing of blood collection was staggered between groups to account for circadian changes in CORT. The supernatant was then flash frozen on dry ice and stored at −80°C until analysis. CORT levels were measured using a Corticosterone ELISA kit (ab108821, Abcam, Cambridge, UK) according to the manufacturer's instructions.

Serum LPS was measured using the Limulus Amoebocyte Lysate (LAL) method according to established protocols (de La Serre et al. 2015). For the LPS measure, all procedures were conducted using endotoxin‐free supplies, and surfaces were thoroughly cleaned with 70% ethanol. Briefly, samples were thawed to room temperature, vortexed for 1 min, diluted 1:5 with pyrogen‐free water (LAL Reagent Water, Lonza, Basel, Switzerland), and vortexed for another minute. Samples were held in a 70°C water bath for 10 min, cooled, and diluted 1:5 again for a final dilution of 1:10. Standards were prepared using Control Standard Endotoxin (#EC010‐5, Associates of Cape Cod Inc., East Falmouth, MA, USA) and pyrogen‐free water. Lastly, a Pyrochrome solution was made by adding Glucashield Buffer to Pyrochrome Lysate Mix (CG1500‐5, Associates of Cape Cod Inc). Samples and standards were plated in an endotoxin‐free 96‐well plate (Pyroplate, Associates of Cape Cod Inc). The Pyrochrome solution was added to each well, and the plate was covered in foil and placed on a heat block at 37°C for 75 min. The absorbance was read at 405 nm every 5 min to obtain the optimal standard curve with a SpectraMax M3 spectrophotometer (Molecular Devices, San Jose, CA, USA). At 75 min, 50% acetic acid was added as the stop solution, and absorbances were read one final time.

For serum melatonin and cytokine analyses, animals were anesthetized with an intramuscular injection of a cocktail of ketamine (90.1 mg/kg body weight), xylazine (2.8 mg/kg body weight), and acepromazine (0.72 mg/kg body weight), and then rapidly decapitated. Trunk blood was collected and allowed to clot on ice for 30 min, before being centrifuged for 10 min at 4°C. Timing of blood collection was staggered between groups to account for circadian changes in melatonin. The supernatant was then flash frozen on dry ice and stored at −80°C until analysis. Melatonin levels were determined using the MILLIPLEX MAP Rat Stress Hormone Magnetic Bead Panel (RSHMAG‐69K; MilliporeSigma, Burlington, MA, USA) by following the manufacturer's protocol. The standards and samples, diluted at 1:4 with assay buffer, were freshly prepared and run in duplicate. Serum cytokines were measured using a custom ProcartaPlex Assay (PPX‐04; ThermoFisher Scientific, Waltham, MA, USA) for TNF‐ α, IL‐1 β, and IL‐10. The standards and samples were diluted 1:4 with assay buffer and run in triplicate. For both melatonin and cytokine measures, the Luminex plates were read on Bio‐Rad Bio‐Plex 200 Systems with Manager Software (Hercules, CA, USA) for data acquisition and analysis.

### 2.5 Immunohistochemistry (IHC) and Neuroinflammation Quantification

IHC analyses were conducted in Cohorts 1 and 2. Animals were anesthetized at 6 weeks post‐surgery via CO_2_ inhalation and were transcardially perfused with PBS and 4% PFA in PBS. Whole brains were collected and post‐fixed in 4% PFA in PBS on ice for 2 h. Brains were transferred to 30% sucrose, 0.1% NaN3 in PBS for at least 24 h. Brains were then frozen at −80°C until further analysis. Brains were coronally sectioned at 20 μm thickness using a Leica CM1900 cryostat and mounted on negatively charged slides. Sections were collected between bregma 3.2 and 2.2 mm for prefrontal cortex and −2.3 to −5.6 for amygdala and hippocampus. Tissue sections were stained for ionized calcium binding adaptor molecule‐1 (Iba1), a marker of activated microglia and macrophages, and glial fibrillary acidic protein (GFAP), which is expressed by astrocytes and upregulated in reactive astrocytes. Sections were permeabilized with sodium borohydride and incubated overnight with primary antibody against Iba1 (anti‐IBA‐1 rabbit, Wako, Cat#019‐19741) and GFAP (anti‐GFAP rabbit, Abcam, AB7260). Subsequently, sections were incubated for 2 h with Alexa 488 secondary antibody (Invitrogen, #A11008). Slides were examined using a Keyence BZ‐X800 fluorescent microscope (Keyence, Osaka, Japan) at 20× magnification. Images were analyzed using QuPath software (Bankhead et al. 2017) for cell counts.

**FIGURE 2:**
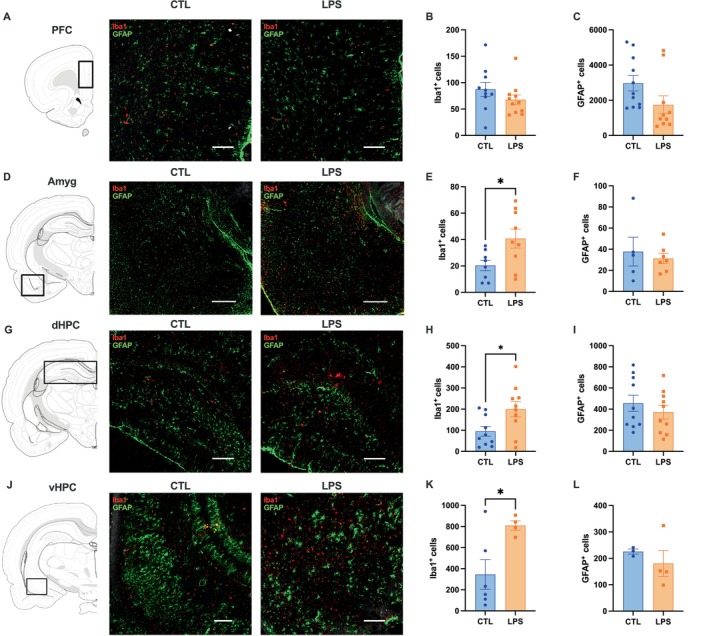
Chronic LPS administration induces neuroinflammation in brain regions associated with learning and memory, impulse control, and anxiety‐like behavior. (A) Representative images for the prefrontal cortex (PFC), showing control (left) and LPS (right) (Scale bar, 200um). Chronis LPS administration does not affect Iba1 or GFAP staining in the PFC (B, C), nor does it affect GFAP staining in the amygdala (Amyg), dorsal or ventral hippocampus (dHPC; vHPC) (F, I, L). However, Iba1 staining was significantly increased in the Amyg, dHPC, and vHPC (E, H, K) in animals receiving chronic low‐dose LPS administration. Representative images for the amygdala, dorsal and ventral hippocampus showing control (left) and LPS (right) are shown as well (D, G, J) (Scale bar, 200um). (LPS *n* = 4–10 CTL *n* = 3–11; all between subjects design; data are means ± SEM; *p < 0.05).

IHC to obtain representative images for Figure 2 were conducted using slightly different protocols than quantification, as the two procedures were conducted in different laboratories. For representative images, brains were sectioned at 30 μm thickness on a freezing microtome. Sections were collected in 5 series and stored in antifreeze solution at −20°C until further processing. General fluorescence IHC labeling procedures were performed. The following antibodies and dilutions were used: rabbit anti‐Iba1 (1:1000, Wako, Cat. # 019‐19741) and chicken anti‐GFAP (1:2000, Cat. # ab4674, Abcam, Cambridge, UK). Antibodies were prepared in 0.02 M potassium phosphate‐buffered saline (KPBS) solution containing 0.2% bovine serum albumin and 0.3% Triton X‐100 at 4°C overnight. After thorough washing with 0.02 M KPBS, sections were incubated in secondary antibody solution. All secondary antibodies were obtained from Jackson ImmunoResearch and used at 1:500 dilution at 4°C, with overnight incubations (Jackson ImmunoResearch; West Grove, PA, USA). Sections were mounted and cover slipped using 50% glycerol in 0.02 M KPBS and the edges were sealed with clear nail polish. Photomicrographs were acquired using either a Nikon 80i (Nikon DS‐QI1,1280X1024 resolution, 1.45 megapixel) under epifluorescence or darkfield illumination. Images were analyzed using ImageJ (Schneider et al. 2012) for cell counts.

### 2.6 Statistical Analysis

All statistical analyses were conducted using GraphPad Prism 10.0.3 (GraphPad Software Inc. Boston, Massachusetts, USA). Data are reported as mean ± SEM. *t*‐tests were used to analyze MWM probe test, NLR, NOIC, OFT, DRL, PR, serum LPS, CORT, melatonin, cytokines, and immunohistochemistry results. Mixed effects analysis followed by Fisher's LSD post hoc analysis for multiple comparisons was used to analyze body weight, food intake, and MWM training. Simple linear regression was used to analyze the relationship between serum LPS levels and behavioral outcomes in the MWM, NLR, and OFT tasks. Statistical significance was set at a *p*‐value ≤ 0.05.

## 3 Results

### 3.1 Chronic LPS Administration Reduces Serum Corticosterone and Melatonin Without Influencing Long‐Term Food Intake or Body Weight

There were no differences in body weight between groups at the start of the experiment. Analysis of body weight showed an expected main effect of time (*F* (1.425, 29.50) = 662.8, *p* < 0.0001) and a significant interaction between time and treatment (*F* (24, 497) = 2.547, *p* < 0.0001) (Figure 1B). In both groups, body weight increased with time (Figure 1B). Following surgery and pump implantation, the LPS group lost significantly more weight than the control group (Days 7–13, *p* < 0.05), which corresponds to the beginning of LPS release (Figure 1B). However, following recovery (1‐week post‐surgery), there were no significant differences in body weight between groups (Figure 1B). There was an overall effect of time (*F* (5.314, 110.6) = 46.50, *p* < 0.0001) on food intake, with both groups decreasing intake following surgery. We also observed a significant time by treatment interaction (*F* (11, 229) = 18.84, *p* < 0.0001) (Figure 1C). LPS‐treated rats exhibited significantly lower food intake compared to control rats following surgery (Days 4–7) which corresponds to the beginning of LPS release (*p* < 0.0001), followed by a period of significantly increased intake (*p* < 0.05) (Figure 1C). However, at the time of behavioral testing, LPS‐treated animals did not significantly differ from their saline counterparts in either body weight or food intake (Figure 1C).

As expected, serum LPS was significantly higher in LPS‐treated animals compared to control animals (*p* < 0.05) (Figure 1D). Results also reveal that LPS‐treated animals had significantly lower levels of serum corticosterone (*p* < 0.05; Figure 1E) and melatonin (*p* < 0.05; Figure 1F) compared to their saline counterparts, suggesting a potentially altered stress response in these animals.

### 3.2 Chronic LPS Administration Induces Neuroinflammation in Brain Regions Associated With Anxiety, Impulsivity, and Learning and Memory

Microglial recruitment and astrocytic reactivity were measured using Iba1 and GFAP immunostaining, respectively, with representative images of regions of interest shown in Figure 2A,D,G,J. We analyzed positive cell counts in the dorsal hippocampus (dHPC), ventral hippocampus (vHPC), prefrontal cortex (PFC), and amygdala (Amyg), as these are brain regions involved in learning and memory (Hayes, Lauer, et al. 2024), impulsivity (Noble et al. 2019), and anxiety‐like behavior (Jimenez et al. 2018). Notably, obesity is linked with impaired learning and memory, as well as altered anxiety and impulsivity (Kanoski and Davidson 2011; Fulton et al. 2022; Giel et al. 2017). Results revealed that there were significantly more Iba1^+^ cells in the Amyg (*p* < 0.05; Figure 2E), dHPC (*p* < 0.05; Figure 1H), and vHPC (p < 0.05, Figure 2K) of LPS‐treated animals compared to controls, while there were no differences between groups in the PFC (*p* = 0.6, Figure 2B). There were no observed differences in GFAP staining in any of the regions of interest that we examined (Figure 2C,F,I,L). Taken together, these results suggest that chronic low‐grade inflammation drives neuroinflammation by increasing microglial activity in brain regions associated with neurocognitive processes.

### 3.3 Chronic LPS Administration Has no Effect on Hippocampal‐Dependent Learning and Memory

**FIGURE 3:**
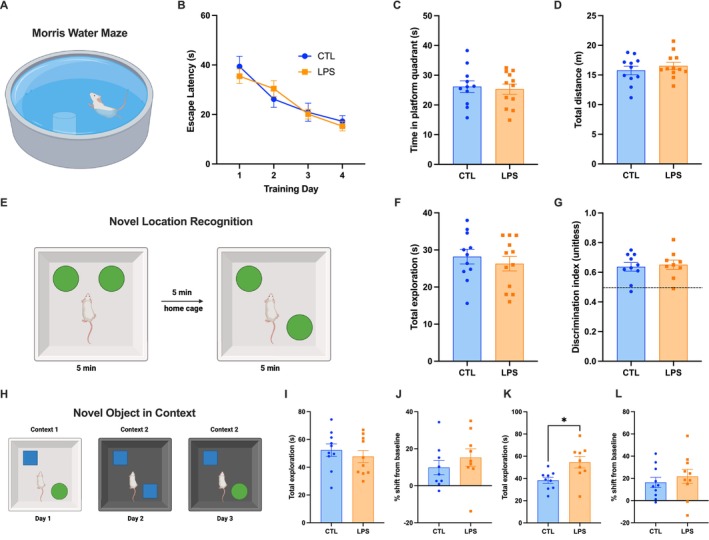
Chronic LPS administration does not affect hippocampal‐dependent memory function. Hippocampal‐dependent learning and memory was unaffected by chronic low‐dose LPS administration in either the Morris water maze (MWM) (A–D) or the novel location recognition (NLR) (E–G). Hippocampal‐dependent memory in the novel object in context (NOIC) task (H) was not affected when tested either early (J) or late (L) in the LPS administration timeline. Total exploration of both objects was significantly increased with chronic LPS administration only at the second testing of the NOIC task (I, K) (LPS *n* = 12, CTL *n* = 11 for MWM, NLR; LPS *n* = 10, CTL *n* = 10 for NOIC; between‐subjects for all experiments; data are means ± SEM; *p < 0.05).

Due to the increased neuroinflammation in the dHPC and vHPC of LPS‐treated animals, and the strong connection between hippocampal‐dependent memory impairments and obesity (Kanoski and Davidson 2011; Noble et al. 2017; Hayes, Lauer, et al. 2024), we tested animals in a variety of hippocampal‐dependent memory tasks. During the Morris water maze (MWM) task, the amount of time to find the platform (escape latency) was measured for each of 5 trials on 4 consecutive training days to assess hippocampal‐dependent learning. The average daily escape latency was calculated for each animal. There was an overall effect of time (*F* (2.686, 54.62) = 32.39, *p* < 0.0001), with average escape latency decreasing over time, indicating that rats in both groups learned to find the platform more quickly as training progressed (Figure 3B). However, there were no differences in performance between groups (Figure 3B). Memory was also assessed during a probe test, and no differences between groups in time spent in the platform quadrant (Figure 3C) or total distance traveled were observed (Figure 3D). Furthermore, there were no significant correlations between LPS serum levels and behavioral outcomes in this task (Figure S1A,B).

We also used the novel location recognition (NLR) and novel object in context (NOIC) tasks to assess hippocampal‐dependent learning and memory. For NLR, there were no differences between groups in exploratory behavior (Figure 3F) or discrimination for the novel object (Figure 3G), nor were there significant correlations between serum LPS levels (Figure S1C). When tested shortly after pump implantation in the NOIC test (21–23 days post‐surgery), animals showed no differences in total exploration of objects or preference for the novel object (Figure 3I,J). However, when retested in NOIC several weeks later with new objects (37–39 days post‐surgery), LPS‐treated animals spent significantly more time exploring both objects when compared to controls (*p* < 0.05; Figure 3K), though there were no differences in the preference for the novel object as measured by percent shift from baseline (Figure 3L). Together, these data suggest that obesity‐ and Western diet‐associated memory impairments from previous reports are unlikely to be driven by chronic low‐grade inflammation or neuroinflammation, or at least not that induced by LPS‐mediated endotoxemia.

### 3.4 Chronic LPS Administration Reduces Anxiety‐Like Behavior and Impulsive Responding for Palatable Foods, Independently of Food Motivation

While learning and memory are unaffected by chronic LPS administration, the brain regions with increased microglial activity shown in Figure 2 are also associated with impulsive responding (Noble et al. 2019) and anxiety‐like behavior (Jimenez et al. 2018). Given these previous findings and the significant difference in exploratory behavior between LPS‐treated and control animals in the NOIC task, animals were tested in the open field test (OFT) and zero maze (ZM) test to assess exploratory and anxiety‐like behavior.

**FIGURE 4:**
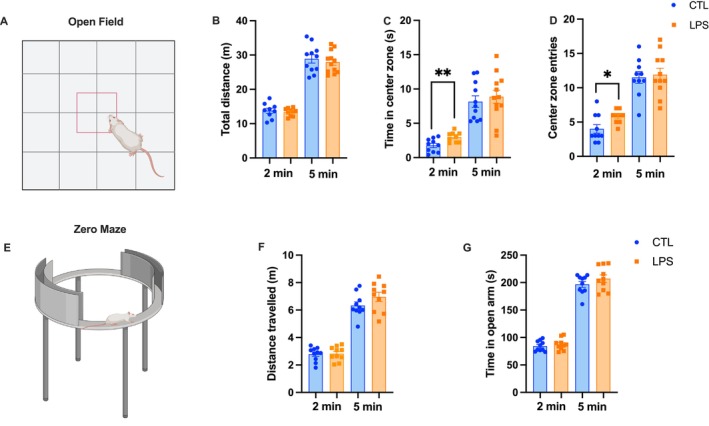
Chronic LPS administration decreases anxiety‐like behavior. Anxiety‐like behavior was significantly decreased in the open field test (A) with chronic LPS administration when compared to controls, with experimental rats spending more time (C) and making more entries into the center zone (D) when compared to control rats at the 2 min timepoint. Total distance traveled was unaffected (B). In the zero maze test (E), both total distance traveled (F) and percent time spent in the open arm of the apparatus (G) were unaffected. (LPS *n* = 9, CTL *n* = 10 for open field, LPS *n* = 10, CTL = 10 for zero maze; all between‐subjects design; data are means ± SEM; *p < 0.05, **p < 0.01).

No differences were seen in behavior during the OFT (Figure 4A) when all 5 min were analyzed (Figure 4A,C,D). However, when behavior during the first 2 min of OFT was analyzed to capture the initial response to a new environment, LPS‐treated rats made significantly more entries into the center zone and spent significantly more time there when compared to control animals (*p* < 0.05; Figure 4C,D), indicating a decrease in anxiety‐like behavior and an increase in exploratory behavior. There were no differences between groups in total distance traveled at either 2 or 5 min (Figure 4B). However, when anxiety‐like and exploratory behavior were assessed via the ZM test (Figure 4E), no differences were seen in total distance traveled or percent time spent in the open arm of the apparatus at either timepoint (Figure 4F,G).

**FIGURE 5:**
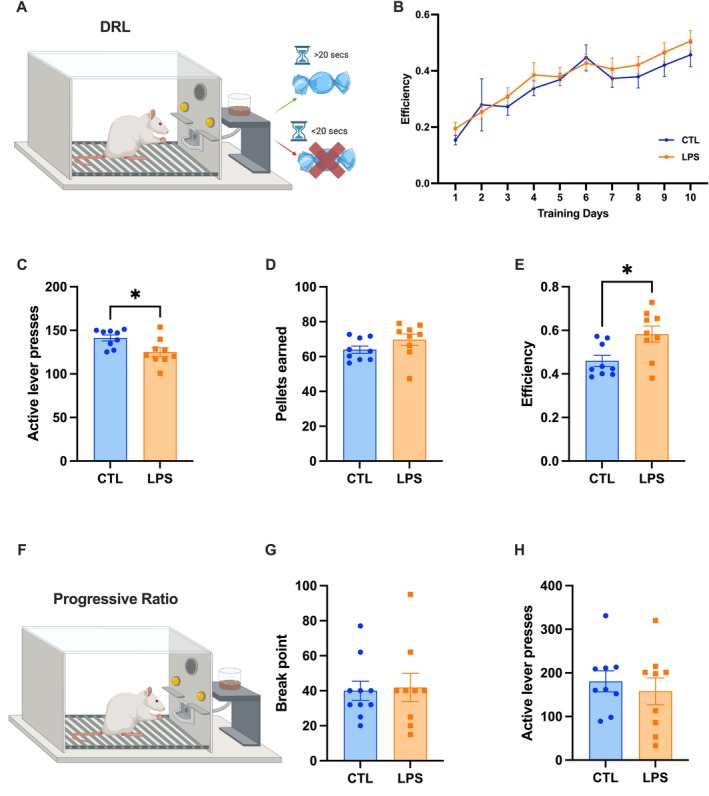
Chronic LPS administration decreases impulsive responding for palatable foods independently of reward motivation. In the DRL test of impulsive action (A), animals were trained to wait at least 20s between lever presses in order to obtain a reward over the course of 10 days (B). Results reveal that animals receiving chronic LPS administration made significantly fewer responses on the active lever (C), while earning the same number of rewards (D), leading to an increase in efficiency in the task (E) when compared to controls. Reward motivation as measured by the progressive ratio (PR) task (F) was unaffected, with both break point (G) and total active lever presses (H) remaining unaffected. (LPS *n* = 9 CTL = 9–10; all between‐subjects design; data are means ± SEM; *p < 0.05).

To assess whether impulsive responding is affected by LPS‐mediated chronic inflammation, animals were tested in the DRL test (Figure 5A) to measure impulsive action. While there was no difference in overall learning during training (Figure 5B), LPS‐treated rats made significantly fewer presses on the active lever while earning the same number of rewards when compared to control rats on test day (Figure 5C,D). This led to LPS‐treated rats having significantly higher efficiency in the task when compared to control animals (as measured by rewards earned over total active lever presses), indicating decreased impulsive responding (*p* < 0.05; Figure 5E).

However, given that a decrease in responses on the active lever could indicate an overall difference in motivation to work for the reinforcement, animals were also tested in the progressive ratio (PR) operant task in which each successive reinforcer earned requires more lever presses than the previous reinforcer (Figure 5F), which is a common test of motivation to work for palatable food. There were no group differences in active lever presses as assessed in PR, nor were there any differences in the final breakpoint between LPS‐treated and control rats (Figure 5G,H), suggesting that the effects on impulsive responding are not secondary to alterations in reward motivation.

## 4 Discussion

Obesity, Western diet consumption, and the concomitant increased gut permeability can lead to metabolic endotoxemia driving both chronic peripheral and central inflammation (Cani et al. 2007; Fuke et al. 2019; Pendyala et al. 2012; Guillemot‐Legris and Muccioli 2017). These factors independently are each associated with neurocognitive deficits, affecting cognitive domains such as anxiety, reward motivation, and learning and memory (Cordner and Tamashiro 2015; Kanoski and Davidson 2011; Francis and Stevenson 2013; Fulton et al. 2022; Wallace and Fordahl 2022; Shi et al. 2022). Thus, disentangling the causal relationships between these variables is increasingly difficult. Here, we modeled the chronic low‐grade inflammation associated with obesity and/or Western diet consumption, yet in nonobese animals maintained on a healthy diet. Metabolic endotoxemia and its associated inflammatory profile were modeled using an indwelling mini osmotic pump to continuously dispense LPS solution. Our results reveal that low‐dose LPS treatment over the course of 6 weeks is sufficient to significantly increase microglial recruitment in the amygdala (Amyg) and hippocampus (HPC; in both dorsal and ventral subregions), reduce serum corticosterone and melatonin, and decrease anxiety‐like and impulsive behaviors. LPS treatment did not, however, affect astrocytic reactivity, HPC‐dependent memory, or food reward‐motivated operant responding.

Previous research indicates that obese individuals have higher levels of circulating LPS relative to healthy weight individuals (Gnauck et al. 2016), although the precise levels of serum LPS can differ greatly between studies (Gnauck et al. 2016). Some studies have considered LPS levels of 0–0.1 ng/mL to be physiologically relevant in mice (Guo et al. 2013), and serum LPS levels in our study fell within this range. One previous study revealed that human participants with obesity have roughly 1.6× higher LPS levels relative to their healthy weight counterparts (Trøseid et al. 2013), which is in line with the difference between saline‐ and LPS‐treated animals in the present study. Thus, the present model appears to approximate physiological levels of LPS in circulation in obesity. However, obesity and Western diet consumption increase inflammatory cytokine signals in addition to LPS (Erion et al. 2014). LPS treatment in the present study led to a trend towards increased TNF‐α and IL‐1β expression in serum when compared to controls, though these differences did not reach statistical significance (Figure S2). Thus, while our LPS model only partially mirrors the chronic inflammation present in obesity, it is consistent with previous research showing that DIO‐associated cytokine elevations relative to healthy controls do not reach levels seen in genetic models of obesity (Bedoui et al. 2005).

Consistent with a previous study using the same dose of LPS in adult rats (de La Serre et al. 2015), LPS treatment in the present study produced an initial hypophagia and body weight loss, followed by a period of hyperphagia and weight regain until body weights were comparable to controls. While a period of hyperphagia without concomitant increases in body weight may indicate the presence of a low‐grade fever (Rummel et al. 2016), the body weights of LPS‐treated rats never exceeded those of controls, thus indicating that excessive body weight is not a characteristic of this model in rats. However, a previous study using adult mice and the same dose of chronic LPS revealed that experimental mice weighed significantly more than controls after 4 weeks of administration (Cani et al. 2007). Thus, while there may be species differences in the effects of chronic low‐dose LPS with regard to body weight effects, results from the present study clearly model elevated LPS in circulation in the absence of obesity, and within a physiological range with regard to obesity.

Changes in behavioral responses may be driven by LPS‐induced neuroinflammation (Zheng et al. 2021; Zhao et al. 2019; Zhang et al. 2022). Chronic LPS exposure significantly increased microglial activation in the Amyg, dHPC, and vHPC, while astrocytic reactivity remained unaffected. Previous literature is consistent with these findings (Zhang et al. 2022; Llorens‐Martín et al. 2014; Hill et al. 2019) and suggests that 6 weeks of LPS treatment is not sufficient time to develop significant alterations in astrocytic activity, but rather, up to 16 weeks of administration may be required to induce astrocytic activity (Schirmbeck et al. 2023). Microglial activation caused by LPS has been previously demonstrated in the HPC (Zhao et al. 2019; Zhang et al. 2022), albeit with doses significantly higher than our own. Similarly, Huang et al. found increases in the size of microglial cell bodies in the basolateral amygdala following 3 acute injections of a supraphysiological dose of LPS (1 mg/kg) 20 weeks prior to Iba1 analyses (Huang et al. 2020). Present results expand these previous findings to suggest that a low physiological dose of LPS when given chronically is sufficient to induce changes in microglia in the HPC and Amyg. These changes in microglia in key brain regions associated with several cognitive domains (i.e., learning, memory, exploratory behavior, food motivation) lead us to investigate possible behavioral outcomes associated with neuroinflammation.

While previous studies suggest that hippocampal‐dependent memory is strongly impacted by neuroinflammation (Barrientos et al. 2015; Czerniawski and Guzowski 2014; de Paula et al. 2021; Ryan and Nolan 2016), learning and memory were unaffected by LPS exposure in our study in the MWM, NLR, and NOIC procedures, despite elevated microglial recruitment in both the dorsal and ventral hippocampus. The lack of differences in learning and memory was surprising, as previous research has shown that LPS exposure can have deleterious effects on memory (Czerniawski et al. 2015; Zhao et al. 2019; Schirmbeck et al. 2023; Valero et al. 2014). These discrepancies could be explained by our study's relatively low dose of LPS (12.5 μg/kg/h) compared to previous studies, as well as by differences in the mode and timing of LPS administration. For example, a majority of previous studies utilized acute injections of a high dose of LPS to induce inflammation, with doses ranging from 167 to 750 μg/mL LPS (Czerniawski et al. 2015; Zhao et al. 2019; Valero et al. 2014). Only one of these studies found deficits in MWM in wildtype animals, using an acute, supraphysiological dose of 500 μg/mL LPS (Zhao et al. 2019). Others found impairments in MWM only in transgenic Alzheimer's disease model mice (Valero et al. 2014) or in NOIC following an acute injection of a high dose of LPS (Czerniawski et al. 2015). Schirmbeck and colleagues, who found impairments in NLR in LPS‐treated rats, did utilize a chronic administration design, although injections took place weekly for 16 weeks and at a significantly higher dose (500 μg/kg) than our own (Schirmbeck et al. 2023). Collectively, our results expand previous literature to suggest that, when chronic endotoxemia is within a physiological range, the learning and memory deficits commonly associated with obesity and Western diet consumption are most likely not driven by peripheral or central inflammation.

Given that the amygdala is heavily implicated in fear and anxiety (Davis 1992), and that we found significant differences in amygdalar neuroinflammation and exploratory behavior in the NOIC procedure, we investigated the effects of LPS‐driven inflammation on established tests of exploratory and anxiety‐like behavior. While acute, high dose (500–830 μg/mL) LPS exposure reliably induced anxiety‐like behavior in previous studies (Bassi et al. 2012; Zheng et al. 2021) the effects of chronic LPS exposure at a physiologically relevant dose are unclear. When measured in the OFT, LPS‐treated animals in the present study spent significantly more time in the center of the arena and made more entries into the center zone when compared to their control counterparts, which is consistent with a previous report (Ryan and Nolan 2016). This could be interpreted as decreased anxiety‐like behavior and increased exploratory behavior, which is consistent with our data from the NOIC procedure revealing more time investigating objects overall in LPS‐ vs. saline‐treated rats. However, the effects of our LPS model on reducing anxiety‐like behavior should be interpreted with caution, as the group differences for OFT were observed at the 2‐min time point, but were not significant at the 5‐min time point. Further, when anxiety‐like behavior was assessed via the ZM task, no differences were found in time spent in the open arm of the apparatus, an outcome consistent with previous findings (Schirmbeck et al. 2023; Loh et al. 2023). There are several possibilities as to why behavior in our study was affected in the OFT but not the ZM task. As metabolic endotoxemia can take up to 4 weeks to fully develop (Cani et al. 2007), it is important to note that our behavioral testing took place around or shortly after this time point, but before the pumps dispense all their solution by 6 weeks. Given this short experimental window for behavioral testing, behavior in the ZM was assessed before the OFT with ZM testing on Day 25 post‐surgery and OF testing 40 days after LPS treatment initiation. Thus, it is possible that the effects of the LPS‐induced chronic inflammation were more robust when behavior was assessed in the OFT vs. the ZM test, which took place shortly before the 4‐week mark from pump implantation. Consistent with this hypothesis, increased overall object exploration time was observed in LPS‐treated rats during the second NOIC memory test (37–39 days post‐surgery), but not during the first NOIC memory test (21–23 days post‐surgery). It is also the case that the OFT and ZM procedures differ in various parameters, including elevation from the floor, temporal length, and test lighting conditions. Nevertheless, our collective results suggest that exploratory behavior is increased, and anxiety‐like behavior is reduced (at least in the OFT) in animals exposed to LPS‐mediated chronic low‐grade inflammation within a physiological range.

The functional connection between circulating LPS levels and anxiety‐like behavior is also supported by our correlational analyses indicating that LPS levels are negatively correlated with entries into the center zone in the OFT task in control rats. Interestingly, LPS levels are positively correlated with entries into the center zone in the LPS‐treated rats, although this trend did not reach statistical significance. Similar effects were observed for distance traveled in the OFT task. While these results were unexpected, it may be the case that circulating LPS influences anxiety‐like behavior in a bidirectional manner, with LPS leading to anxiogenic effects at lower circulating levels and to anxiolytic effects at higher circulating levels. Additional experiments are required to assess this hypothesis.

Increased entries into the center zone in OFT are often interpreted as decreased anxiety, but this behavior may also be driven by increased impulsivity. Therefore, we assessed the effects of chronic LPS exposure on impulsive responding for palatable foods. Results revealed that impulsivity for palatable food‐reinforced responding was also altered by LPS treatment, with experimental animals being significantly more efficient in earning rewards in the DRL test of impulsive action when compared to controls (interpreted as less impulsivity). While LPS‐exposed rats did make significantly fewer presses on the active lever in the DRL task, we found no differences in the number of active lever presses or the breakpoint threshold when assessed in the operant PR task. This suggests that our findings in DRL are unlikely to be based solely on overall difference in food reward motivation, and that chronic low‐grade inflammation driven by LPS exposure is sufficient to decrease impulsivity. Previous research suggests that LPS exposure can have a dampening effect on reward‐motivated behavior (Vichaya et al. 2014; Lasselin et al. 2017; De La Garza et al. 2005; Huwart et al. 2024) and increase impulsive responding (Gruzdeva et al. 2021; Straley et al. 2017; Russell et al. 2022), though differences in dose, mode, and timing of LPS administration could all play a part. For example, Lasselin and colleagues found differences in reward behavior in the Effort Expenditure for Rewards Task in humans following a single LPS injection, with LPS driving healthy individuals to button press less frequently for high‐effort tasks (Lasselin et al. 2017). However, this effect was related to sleepiness, with LPS increasing sleepiness significantly 3 h post‐injection, and no differences in reward sensitivity were found (Lasselin et al. 2017). Similarly, male wildtype mice made significantly fewer nose pokes in a low‐effort, low‐reward vs. high‐effort, high‐reward choice task following a single injection of a supraphysiological dose of LPS (33 mg/kg) 24 h prior to testing (Vichaya et al. 2014). Thus, these previous findings taken together with present results suggest that while acute high doses of LPS can reduce reward‐motivated responses, the effects of chronic physiological LPS treatment have minimal impact on motivated behavior, and can even reduce reward‐driven impulsivity.

Regarding impulsivity, previous findings showing that LPS increased impulsive responding may also be explained by acute versus chronic administration, as well as other contributing factors. In several studies, acute injections were given early in life (Gruzdeva et al. 2021; Straley et al. 2017), during developmental time periods that are known to be particularly sensitive to environmental insults (Hoffman et al. 2021; Van den Bergh et al. 2020). While Russell and colleagues found elevated premature responding in the 5‐choice serial reaction time test in LPS‐treated animals, acute injections took place immediately prior to behavioral testing and at a dose intended to cause sickness behavior (150 μg/mL) (Russell et al. 2022). Thus, it is unsurprising that there are behavioral differences between their study using a supraphysiologic dose and the present findings. Collectively, our results expand previous literature by revealing that while acute and high dose LPS treatment may reliably reduce reward‐motivated responses and increase impulsivity, chronic endotoxemia‐induced inflammation within a physiological range can have the opposite effect.

It is possible that our behavioral results may be explained, in part, by differences in stress responsivity between LPS‐ and saline‐treated animals, as serum corticosterone and melatonin were both significantly decreased in LPS‐treated experimental animals when compared to controls. While previous research has shown that LPS increases serum corticosterone levels, it is likely due to the acute injection of a high dose of LPS (1.5 mg/kg) prior to serum collection (Girard‐Joyal et al. 2015). The hypothalamic–pituitary–adrenal (HPA) axis, which plays a crucial role in mediating responses to stress, is known to exhibit bidirectional effects on cortisol levels (Rutters et al. 2012; Rusch et al. 2023), and previous research suggests that stressors can exert negative feedback effects on HPA axis activity by suppressing corticotropin‐releasing hormone (CRH) neuron activity (Jiang et al. 2022). Additionally, cortisol responses to LPS injections are dampened in individuals with obesity (Lasselin et al. 2020). These results are in line with our current study, suggesting that altered HPA axis activity may be implicated in behavioral changes associated with chronic inflammation (Rutters et al. 2012; Rusch et al. 2023; Packard et al. 2016).

Melatonin is also known to inhibit neuroinflammation (Wang et al. 2022) and plays an important role in HPA axis regulation. The current study found that serum melatonin was reduced in LPS‐treated rats when compared to those receiving saline. Previous research has also suggested a role for melatonin in inflammation‐induced cognitive changes. Papp et al. found that acute injections of melatonin enhanced open arm exploration in the elevated plus maze task (Papp et al. 2006), suggesting a possible role for melatonin in reducing anxiety‐like behavior. Importantly, previous research also suggests that melatonin acts to attenuate the degradation of tight‐junction proteins caused by LPS treatment (Hu et al. 2017) and that anxiety induced by an acute injection of a high dose of LPS (250 μg/kg) 3 h before testing is attenuated with concomitant melatonin injections in rats (Nava and Carta 2001). These results show a potentially important role for melatonin in the modulation of LPS‐mediated inflammation‐induced cognitive changes. However, the seemingly complex interaction between LPS and melatonin signaling, as well as how acute vs. chronic LPS may differentially impact melatonin levels and anxiety‐like behavior, requires additional mechanistic follow‐up studies. Indeed, these previous studies highlight a negative relationship between melatonin and anxiety‐like behavior (increased melatonin reduces anxiety), whereas present results indicate the opposite relationship.

In the current study, only males were used to study the neurobiological and behavioral effects of chronic LPS administration. There is evidence of sex differences in inflammatory responses (Pitychoutis et al. 2009; Santos‐Galindo et al. 2011), making this a significant limitation of the present study. It has been shown that estrogen can have a protective effect against Western diet‐induced hippocampal dysfunction (Christensen et al. 2020; Pratchayasakul et al. 2011; Pratchayasakul et al. 2015; Pyter et al. 2013), and several studies reveal sex differences in neurocognitive responses to a Western diet (Tsan, Sun, et al. 2022; Hayes, Kao, et al. 2024). Additionally, sex differences are also present in impulsive responding, with males exhibiting higher trait impulsivity than females (Weafer and de Wit 2014). Given the protective effects of estrogen on diet‐associated hippocampal dysfunction, and that females exhibit lower baseline levels of impulsivity than males, we chose males as a starting point for evaluating this model. It is important for future research to explore whether chronic low‐dose LPS administration has similar effects in females.

Here, our collective results support a model in which elevated serum LPS caused by chronic exposure to a physiologically relevant dose of LPS, and the concomitant peripheral and central inflammation, leads to a dampened HPA axis response and hyposensitivity to stress. This HPA axis dysregulation then results in decreased serum corticosterone and melatonin, as well as reduced impulsive responding and anxiety‐like behavior. Importantly, these endotoxemia‐mediated physiological and behavioral changes in the absence of obesity or Western diet consumption do not appear to mediate the widely established effects of both obesity and Western diet on the dysregulation of hippocampal‐dependent memory. Future research should investigate the effects of chronic low‐dose LPS exposure on HPA axis reactivity to identify precise mechanisms linking chronic inflammation to behavioral outcomes, as well as to better understand the effects of acute vs. chronic inflammation on neurocognition when inflammatory treatments are kept within a physiological range. Further, it is important to follow up on what, if not neuroinflammation, mediates Western diet and obesity‐associated learning and memory impairments.

## Conflicts of Interest

The authors declare no conflicts of interest.

## Supporting information


**Figure S1:** LPS levels are associated with changes in performance in the open field test. LPS serum levels are not associated with changes in behavior in the Morris water maze (A, B) or novel location recognition (C) tests. Furthermore, there was no association between serum LPS levels and time spent in the center zone in the open field test (OFT) (D). Analyses reveal a significant correlation between serum LPS and number of entries (E) and distance traveled (F) in the OFT for CTL animals.

**Figure S2:** Inflammatory cytokines are not increased with chronic LPS administration. Serum TNF‐α (A), IL‐β (B), and IL‐10 (C) levels are not significantly affected by long‐term LPS administration.

## Data Availability

The data that support the findings of this study are available from the corresponding author upon reasonable request.
