# Supplementary figures and images for: Endotoxemia‐Induced Inflammation in the Absence of Obesity Is Associated With Decreased Anxiety‐Like and Impulsive Behavior With no Effect on Learning and Memory
Source: Compr Physiol. 2025 Aug 29;15(5):e70044. doi: 10.1002/cph4.70044 (PMC12397682; doi:10.1002/cph4.70044)

# Supplementary Figure 1.

**A**

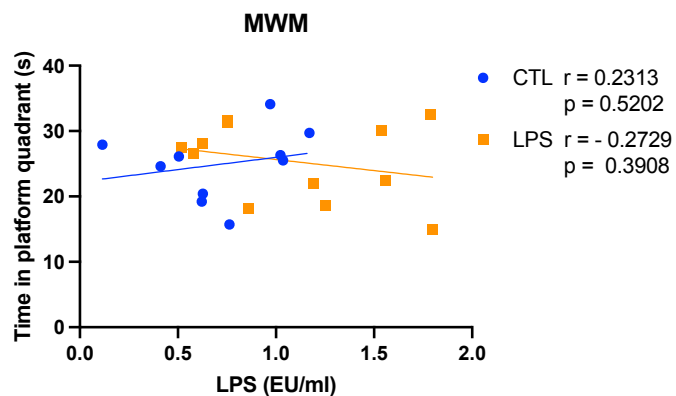

**B**

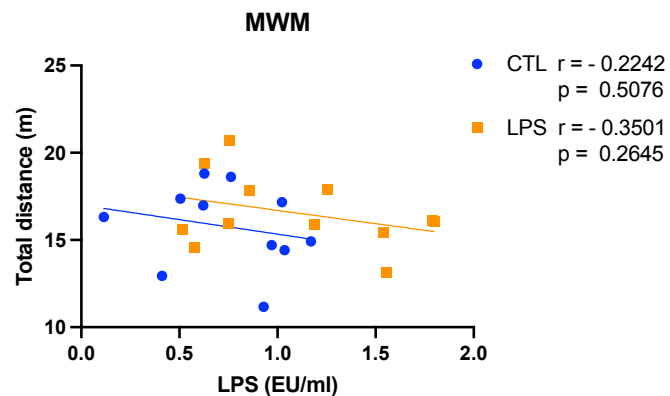

**C**

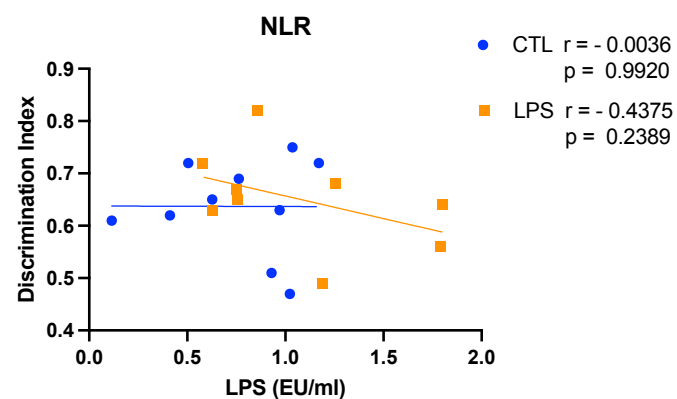

**D**

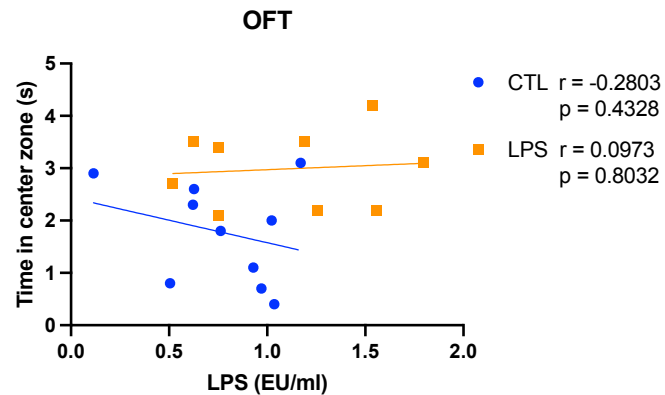

**E**

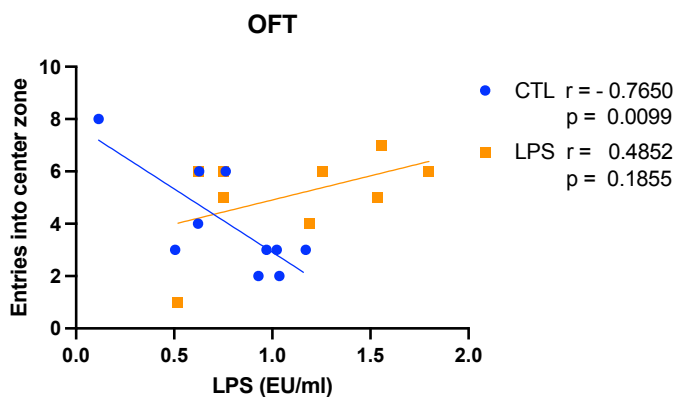

**F**

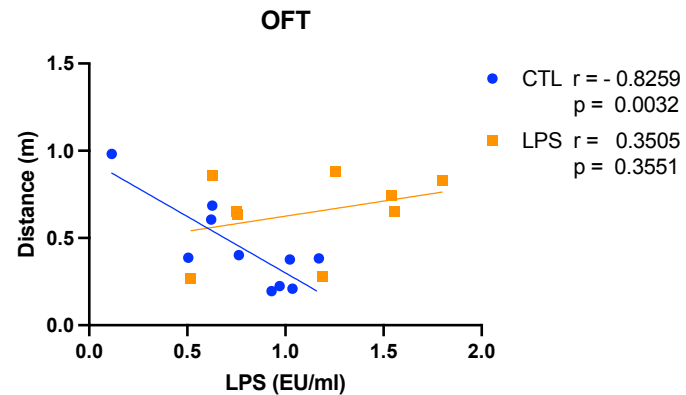

Supplement: Supplementary file 1 — Figure S1: LPS levels are associated with changes in performance in the open field test. LPS serum levels are not associated with changes in behavior in the Morris water maze (A, B) or novel location recognition (C) tests. Furthermore, there was no association between serum LPS levels and time spent in the center zone in the open field test (OFT) (D). Analyses reveal a significant correlation between serum LPS and number of entries (E) and distance traveled (F) in the OFT for CTL animals. [file CPH4-15-e70044-s001.pdf]

Supplementary Figure 2.

A

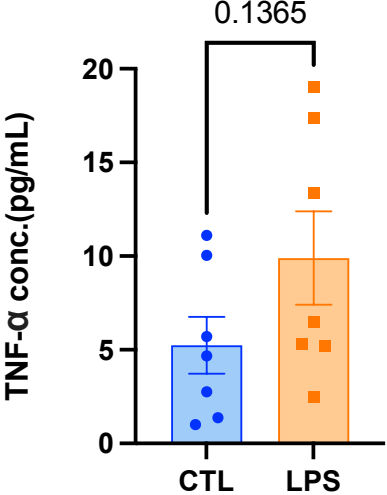

B

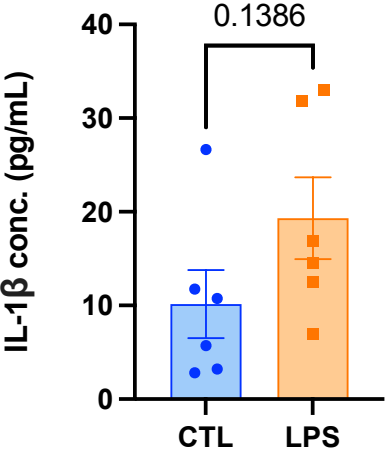

C

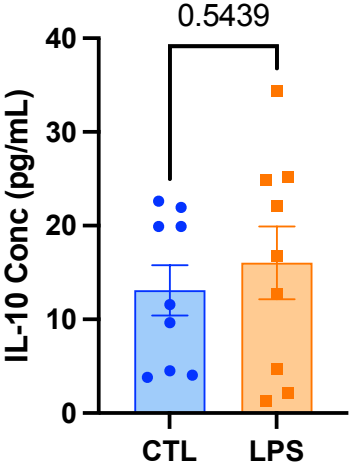

Supplement: Supplementary file 2 — Figure S2: Inflammatory cytokines are not increased with chronic LPS administration. Serum TNF‐α (A), IL‐β (B), and IL‐10 (C) levels are not significantly affected by long‐term LPS administration. [file CPH4-15-e70044-s002.pdf]
